# Electron balancing under different sink conditions reveals positive effects on photon efficiency and metabolic activity of *Synechocystis* sp. PCC 6803

**DOI:** 10.1186/s13068-019-1378-y

**Published:** 2019-02-27

**Authors:** Marcel Grund, Torsten Jakob, Christian Wilhelm, Bruno Bühler, Andreas Schmid

**Affiliations:** 1 Department of Solar Materials, Helmholtz Center for Environmental Research GmbH–UFZ, Permoserstraße 15, 04318 Leipzig, Germany; 2 Plant Physiology Group, Institute for Biology, University of Leipzig, Johannisallee 21-23, 04103 Leipzig, Germany

**Keywords:** Cyanobacterium, Photosynthesis, Electron sink, Electron balance, Quantum efficiency

## Abstract

**Background:**

Cyanobacteria are ideal model organisms to exploit photosynthetically derived electrons or fixed carbon for the biotechnological synthesis of high value compounds and energy carriers. Much effort is spent on the rational design of heterologous pathways to produce value-added chemicals. Much less focus is drawn on the basic physiological responses and potentials of phototrophs to deal with natural or artificial electron and carbon sinks. However, an understanding of how electron sinks influence or regulate cellular physiology is essential for the efficient application of phototrophic organisms in an industrial setting, i.e., to achieve high productivities and product yields.

**Results:**

The physiological responses of the cyanobacterium *Synechocystis* sp. PCC 6803 to electron sink variation were investigated in a systematic and quantitative manner. A variation in electron demand was achieved by providing two N sources with different degrees of reduction. By additionally varying light and CO_2_ availabilities, steady state conditions with strongly differing source–sink ratios were established. Balancing absorbed photons and electrons used for different metabolic processes revealed physiological responses to sink/source ratio variation. Surprisingly, an additional electron sink under light and thus energy limitation was found not to hamper growth, but was compensated by improved photosynthetic efficiency and activity. In the absence of carbon and light limitation, an increase in electron demand even stimulated carbon assimilation and growth.

**Conclusion:**

The metabolism of *Synechocystis* sp. PCC 6803 is highly flexible regarding the compensation of additional electron demands. Under light limitation, photosynthesis obviously does not necessarily run at its maximal capacity, possibly for the sake of robustness. Increased electron demands can even boost photosynthetic activity and growth.

**Electronic supplementary material:**

The online version of this article (10.1186/s13068-019-1378-y) contains supplementary material, which is available to authorized users.

## Background

The photosynthetic machinery is well conserved among phototrophic organisms. Cells are assumed to optimize energy and carbon utilization capacities for efficient biomass formation. Yet, recent studies revealed that only a minor part of the light available and absorbed by the cells is actually used for biomass formation. Even micro-algal cells, which are considered to be more efficient compared to plant cells (compare [1] and references therein), display a poor light efficiency. This is believed to be a consequence of the general sink limitation of photosynthesis when the cells are exposed to a surplus of light [2–8]. The kinetics of biomass formation depends on a rate limiting metabolic step or a developmental process during cell growth. Among others, these can be replication and cell division rates, the carbon fixation capacity, diffusion processes within the cell, or limiting nutrient or energy availability [1, 8]. Any absorbed light energy exceeding the metabolic sink demands or sink utilization capacity has to be dissipated to prevent cellular damage and photoinhibition. Thus, phototrophic microorganisms continuously balance the cellular source/sink ratio of energy assimilation (light harvesting and photosynthetic electron flux) and the metabolic energy demand (mainly for nutrient assimilation and biomass formation) under changing environmental conditions. A phototrophic cell can be forced into an unbalanced state by, e.g., sudden light limitation, excessive irradiance, nutrient limitation, or a combination thereof. Phototrophs possess a number of mechanisms to react to such an unbalanced state on short-term, including non-photochemical quenching and alternative electron quenching pathways as for example Mehler and Mehler-like reactions (reviewed in [9, 10]). On the long term, a new balance is reached mainly by the adjustment of the Chl*a* content, the reduction of PSII rates, the variation of the PSI to PSII reaction rates, the regulation of the cellular RubisCO content, and changes in carbon allocation (reviewed in [2, 4–8]). Cyanobacteria possess a variety of alternative electron sink pathways mediated by, e.g., flavodiiron proteins [11, 12]. This indicates that cyanobacteria strongly rely on alternative electron sinks such as O_2_ to cope with unbalanced and suboptimal growth conditions as described above. Such dissipation of photosynthetic energy restricts the quantum use efficiency of photosynthetic biomass formation.

During recent years, microalgae and particular cyanobacteria gained increasing attention as catalysts for the synthesis of (fine) chemicals and fuels ultimately from CO_2_ in climate friendly processes to replace fossil resources (for comprehensive reviews see [13–16]). In addition, the direct draining of photosynthetically activated electrons derived from water splitting for biotransformations (examples are [17–24]) and ultimately hydrogen production (reviewed in [25, 26]) constitutes an interesting approach for a future bioeconomy. These approaches, however, depend on answers to the following questions: what is the true (biological) potential of phototrophic cells? How can we exploit this potential more efficiently for biomass or product formation? To answer these questions, we need to understand the mechanisms and general metabolic concepts determining the regulation of the light reaction and general cell physiology by a (metabolic) sink. It is widely accepted that photosynthesis is highly sink-limited. Metabolic sinks/processes or nutrient availability are limiting, if light is available in excess [3, 8]. The main hypothesis is that additional sinks have the potential to relief this limitation and unleash higher photosynthetic capacities. It was observed in cyanobacteria that additional electron/carbon sinks can indeed relief such a limitation to a certain extent and result in increased photosynthetic activity/efficiency and even elevated carbon fixation rates. This was shown inter alia for additional carbon sinks in cyanobacteria excreting 2,3-butanediol [27], glycerol [28], sucrose [29, 30], and isoprene [31], but also for the draining of photosynthetic electrons by electron-demanding enzymes [24]. Despite of such findings indicating positive effects of sinks on photosynthesis efficiency, quantitative descriptions in terms of energy balances are not available. The focus so far has been set on the engineering of strains regarding enzymatic/cellular activities, product titer and formation rate, and strain stability. A deeper understanding is missing on how the cells balance metabolic sinks (biomass formation and product formation) with available resources (harnessing of light and photosynthetic electron activation) in the presence of additional sinks.

Nitrate and ammonium constitute two nitrogen (N) sources with strongly differing degrees of reduction. In comparison to ammonium, nitrate requires eight additional electrons per assimilated nitrogen atom and thus represents a significant electron sink [32]. Phototrophs typically prefer the assimilation of ammonium when supplied with both nitrate and ammonium simultaneously [33–35]. Nitrate assimilation, as compared to ammonium assimilation, can thus be expected to have significant effects on cell physiology [36]. Surprisingly, growth rates and quantum yields upon cultivation with ammonium or nitrate as N source did not give a consistent picture such as a higher quantum yield for growth with ammonium as N source (reviewed in [32]). This could be due to secondary effects of ammonium/ammonia, such as photodamage at PSII, decoupling of cyclic and linear electron flow, or global regulatory effects [37–41].

In this study, we compared the assimilation of nitrate and ammonium based on a broad set of physiological parameters and photon/electron balancing [42–45]. This approach was followed to investigate long-term acclimation responses to differing electron demands in the model cyanobacterium *Synechocystis* sp. PCC 6803 (hereafter referred to as PCC6803) and included the analysis of three steady metabolic states based on differing source–sink (light-CO_2_) availabilities. The respective experimental setup involved continuous bioreactor cultivation at low biomass concentrations and constant and defined environmental conditions. Thereby, this study sets a basis for future systematic analyses of electron sink effects on the physiology of PCC6803 and respective underlying regulatory mechanisms.

## Results

### Establishment of different metabolic states in PCC6803 by modulating source and sink availability

This study aimed at the characterization of the physiological responses of PCC6803 to the replacement of the N-source ammonium with nitrate functioning as an additional electron sink. For this purpose, a photon/electron-balancing approach was applied. This approach was used previously to investigate the physiological response and the metabolic electron partitioning in unicellular algae in response to changing environmental conditions [42–45]. For the reproducible characterization of cell physiology via a photon/electron balance, it is important to cultivate the cells under steady state conditions, i.e., constant and controlled environmental conditions, in a well-defined bioreactor setup. This included steady illumination and low turbidity to minimize self-shading. First, the cells were grown in batch mode up to an OD_750_ of approx. 0.5. Then, a feed with fresh growth medium was started and adjusted to maintain a constant turbidity and thus cell concentration (turbidostat) for each experimental condition. The established steady state biomass concentrations did not exceed 120 ± 4 mg_CDW_ l^−1^ (Table 1).

**Table 1:** Basic physiological parameters of PCC6803 under different steady conditions with ammonium or nitrate as N source

	LLHC		HLHC		HLLC	
	NH_4_	NO_3_	NH_4_	NO_3_	NH_4_	NO_3_
*D* (day^−1^)	1.15	1.13	2.45	2.83	0.77	0.74
*X* (mg_CDW_ l^−1^)	107 ± 10	111 ± 7	63 ± 1	58 ± 1	120 ± 4	91 ± 3
*c*(Chl*a*) (mg_Chl*a*_ l^−1^)	2.18 ± 0.29	2.64 ± 0.22	1.50 ± 0.07	1.63 ± 0.10	1.41 ± 0.09	1.05 ± 0.03
Chl*a*_CDW_ (µg_Chl*a*_ mg_CDW_^−1^)	20.4 ± 4.6	23.8 ± 3.5	23.8 ± 1.5	27.7 ± 2.2	11.7 ± 1.1	11.5 ± 1.1
*Y*(PSII) (–)	0.50 ± 0.01	0.60 ± 0.01	0.34 ± 0.01	0.52 ± 0.01	0.16 ± 0.01	0.21 ± 0.03
a*_phy_ (m^2^ g_Chl *a*_^−1^)	15.0 ± 0.8	14.0 ± 0.4	14.5 ± 0.1	15.2 ± 0.2	18.3 ± 1.3	17.9 ± 1.6
*Q*_phar_ (mmol quanta mg_CDW_^−1^ day^−1^)	1.28 ± 0.16	1.36 ± 0.09	6.20 ± 0.37	7.28 ± 0.59	3.56 ± 0.55	3.73 ± 0.38
*r*_F_ (µmol O_2_ mg_CDW_^−1^ day^−1^)	81.5 ± 10.8	102.3 ± 6.6	260.6 ± 23.0	469.4 ± 48.3	68.1 ± 7.4	108.3 ± 11
*r*_O_ (µmol O_2_ mg_CDW_^−1^ day^−1^)	28.9 ± 7.7	34.2 ± 7.9	84.5 ± 16.1	142.8 ± 21.2	39.9 ± 6.4	44.8 ± 4.9
*r*_resp_ (µmol O_2_ mg_CDW_^−1^ day^−1^)	7.7 ± 1.7	13.3 ± 3.0	19.7 ± 3.3	24.3 ± 4.5	5.2 ± 1.0	5.9 ± 0.4
PQ (mol mol^−1^)	1.8 ± 0.1	1.9 ± 0.1	1.5 ± 0.1	1.7 ± 0.1	1.6 ± 0.1	1.6 ± 0.1
C/N (mol mol^−1^)	4.67 ± 0.02	4.69 ± 0.13	4.61 ± 0.01	4.86 ± 0.07	5.23 ± 0.14	5.39 ± 0.14

Mean values and standard deviations correspond to 3–12 samples (depending on the parameter, see “Materials and methods” section) taken during at least three different days for the same metabolic state. LLHC, low light (65 µmol photons m^−2^ s^−1^) and high carbon (1% CO_2_) condition; HLHC, high light (250 µmol photons m^−2^ s^−1^) and high carbon condition (1% CO_2_); HLLC, high light (250 µmol photons m^−2^ s^−1^) and low carbon condition (ambient CO_2_); *D*, dilution rate equaling the specific growth rate *µ* in the respective steady state; *X*, biomass concentration; *c*(Chl*a*), volumetric chlorophyll *a* concentration; Chl*a*_CDW_; biomass-specific Chl*a* content; *Y*(PSII), effective quantum yield at PSII; a*_phy_, Chl*a* dependent absorption coefficient of the cells; *Q*_phar_, rate of quantum uptake; *r*_F_, fluorescence-based electron flux at PSII; *r*_O_, net O_2_ evolution rate; *r*_resp_, respiration rate; PQ, photosynthetic quotient defined by the ratio of O_2_ evolution and CO_2_ uptake; C/N, molar ratio of carbon and nitrogen in biomass

**Fig. 1:**
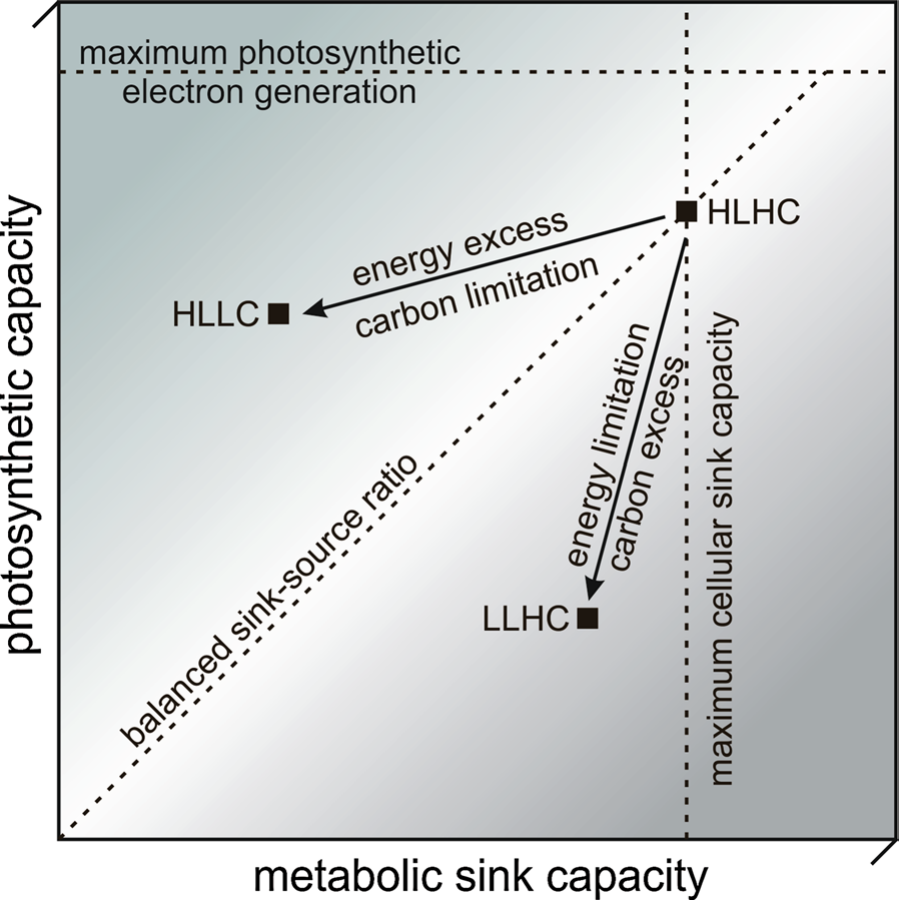
Metabolic states induced by the selected light and carbon dioxide availabilities. In the reference condition HLHC (high light and high carbon), light and carbon are supplied in excess (250 µmol photons m^−2^ s^−1^, 1% CO_2_). The cells can be expected to optimize resource utilization for optimal growth. Under HLHC conditions, growth is restricted by cellular limitations, i.e., saturation of metabolic pathways as for example the Calvin–Benson cycle or other molecular constraints (compare text). Limiting light availability and excess carbon dioxide supply (65 µmol photons m^−2^ s^−1^, 1% CO_2_, LLHC) will force the cells into an electron source-limited metabolic state. In this case, the available light has to be exploited as efficiently as possible to achieve optimal growth. Growth is determined by the light harvesting capacity and PSII efficiency. If light is provided in excess, but carbon availability is limiting (250 µmol photons m^−2^ s^−1^, ambient CO_2_, HLLC), the cells are expected to reduce expenses for photosynthetic source utilization to optimize sink utilization. In both cases, LLHC and HLLC, cells are expected to respond with reduced growth compared to the HLHC reference state

To investigate and quantify possible electron sink effects, three continuous cultivation conditions differing in electron source and sink availabilities were applied: (i) low light (65 µmol photons m^−2^ s^−1^, LL) with excess supply of CO_2_ (1% CO_2_, HC) to induce light and thus source limitation, (ii) high light (250 µmol photons m^−2^ s^−1^, HL) with limiting CO_2_ supply (ambient air, LC) to induce an electron sink limitation, and (iii) unlimited conditions with high light (HL) and excess CO_2_ supply (HC) (Fig. 1). Under the assumption that the cells objective is to maximize growth [46], the cells should operate at maximum light harvesting capacity and quantum efficiency upon light limitation (LLHC). The additional costs for nitrate assimilation should consequently lead to a reduced growth rate as less electrons are available for carbon fixation in the Calvin–Benson cycle. Under HLLC conditions, which force the cells into a carbon dioxide limited state, nitrate as compared to ammonium as N source can be expected not to affect the growth rate, but to cause an increase in light reaction activity to meet the higher electron demand. A similar behavior can be expected for the HLHC condition, which served as a reference condition.

**Fig. 2:**
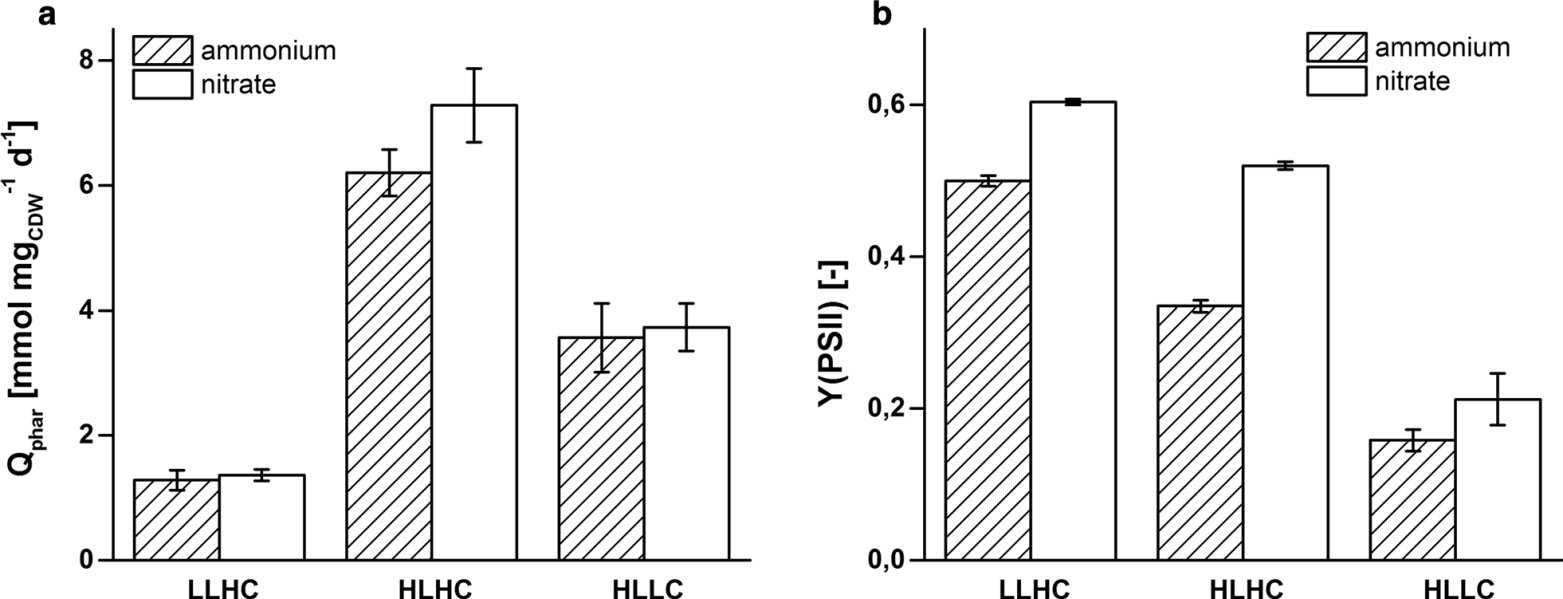
Quantum absorption rates *Q*_phar_ (**a**) and effective quantum yields at PSII *Y*(PSII) (**b**) under different steady state conditions. PCC6803 was grown with ammonium (striped bars) or nitrate (white bars) as N source under LLHC (low light high carbon), HLHC (high light high carbon), and HLLC (high light low carbon) conditions. Cells were cultivated in a bioreactor setup in a continuous (turbidostat) mode to achieve stable steady metabolic states. Light and carbon supply were varied to force the cells into different sink–source balances. The light intensity was set to either 65 (LL) or 250 (HL) µmol photons m^−2^ s^−1^, and carbon was supplied with ambient CO_2_ concentration in air (LC) or air enriched with 1% CO_2_ (HC). Mean values and standard deviations were calculated for three different measurement days. See “Materials and methods” for details on the measurements

Under unlimited HLHC conditions, when cellular processes and capacities are expected to limit growth, a constant growth rate of 2.45 day^−1^ was obtained, which, as expected, was the highest among the three conditions applied (Table 1). LLHC conditions resulted in a 47% reduced growth rate of PCC6803 compared to the reference HLHC condition (Table 1). This indicated that a light limitation was achieved, which was confirmed by the light induction curve (LIC, see Additional file 1: Figure S1) showing that the photosynthetic oxygen evolution rate at the applied light intensity of 65 µmol photons m^−2^ s^−1^ was within the linear alpha slope (Additional file 1: Figure S1A). The specific chlorophyll *a* (Chl*a*) content remained at a similar level as under HLHC conditions (Table 1). The decrease of carbon dioxide supply from 1% to ambient air concentrations under HLLC conditions resulted in a strong decrease of the growth rate by 69% compared to HLHC conditions, which confirms the C- and thus sink limitation. As expected, the biomass-specific Chl*a* content decreased by 50% compared to HLHC (Table 1). The cells decreased their absorption capacity, which also was reflected by the 50% decrease of the rate of absorbed quanta and the severely lower *Y*(PSII) in comparison to HLHC conditions (Table 1 and Fig. 2).

In conclusion, the three selected combinations of light and carbon availability forced the cells into three different sink–source balanced metabolic states. A clear physiological response to sink–source modulation without any effects of fluctuating light and short-term adaption effects was achieved. The following sections describe in detail the impact of replacing ammonium with nitrate as an additional electron sink on the physiology of PCC6803 under these different sink–source balances.

### Effect of an additional electron sink under source limitation

Under source-limited conditions (LLHC), cell growth is restricted by the light availability fueling water splitting and thus the electron transport chain to regenerate NADPH and ATP. Consequently, the cells should possess a maximized light-harvesting capacity and the photosynthetic electron transport should operate at maximum efficiency at the given irradiance. Any additional electron sink is expected to consume electrons at the expense of the electrons available for carbon fixation and finally growth. We tested this hypothesis under steady state LLHC conditions with the supply of nitrate instead of ammonium as N source.

**Fig. 3:**
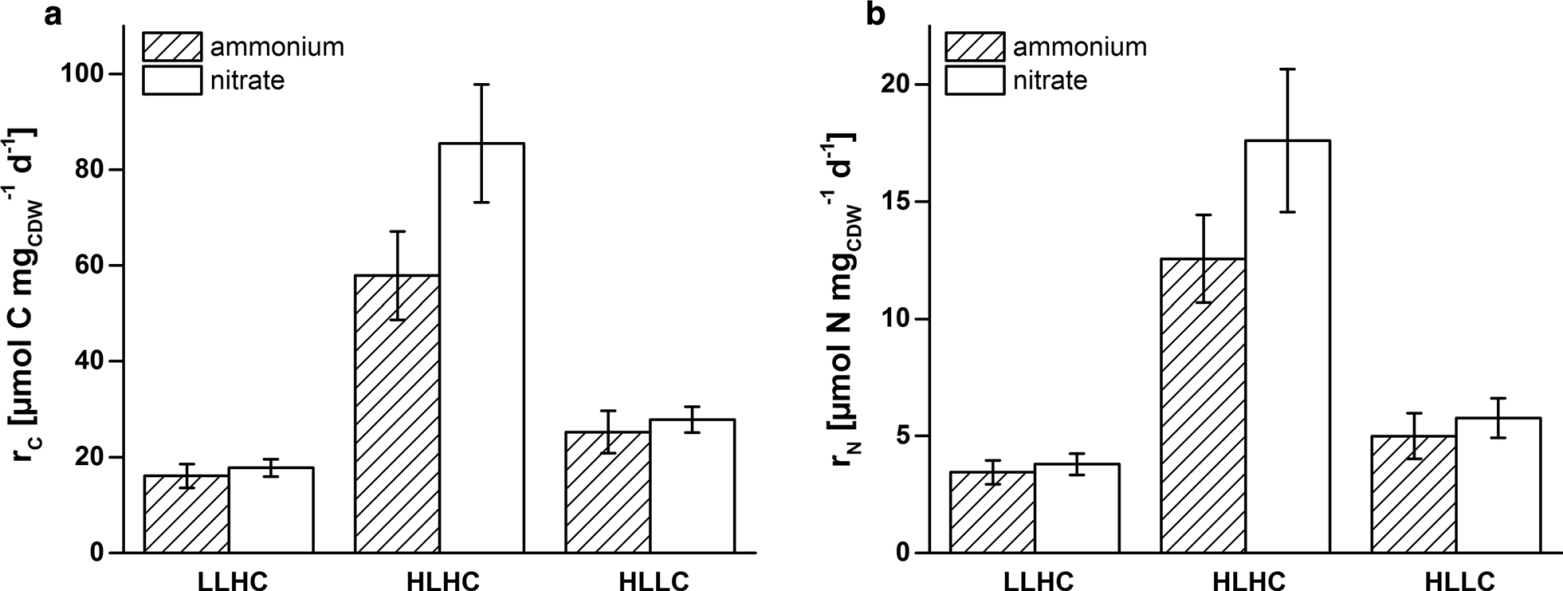
Molar carbon (*r*_C_, **a**) and nitrogen (*r*_N_, **b**) assimilation rates of PCC6803 under different steady state conditions. Cells were grown with ammonium (striped bars) or nitrate (white bars) as N source under LLHC (low light high carbon), HLHC (high light high carbon), and HLLC (high light low carbon) conditions. Growth conditions are described in the legend of Fig. 2. Mean values and standard deviations were calculated for three different measurement days. See “Materials and methods” for details on the measurements

In contrast to the expectations, nitrate instead of ammonium supply did not affect growth and carbon assimilation rates (Table 1 and Fig. 3a). The light harvesting capacity is defined by the Chl*a*-dependent absorption coefficient of the cells (a*_phy_) and finally by the quantum absorption rate of the cells (*Q*_phar_). Cells fed by either nitrate or ammonium did not differ regarding specific Chl*a* content and a*_phy_ (Table 1), resulting in similar quantum absorption rates (Table 1 and Fig. 2a). This met the expectation that cells should display maximum light harvesting capacity in a light-limited regime irrespective of the presence of an additional electron sink. It was further expected that the quantum efficiency *Y*(PSII), i.e., the efficiency of quantum usage at PSII for photochemistry, is maximized in LL and, consequently, should not be influenced by the different electron demand with nitrate and ammonium as N-source. *Y*(PSII), however, increased from 0.50 ± 0.01 to 0.60 ± 0.01, if nitrate instead of ammonium was supplied (Table 1 and Fig. 2a). The increased quantum efficiency in combination with the constant uptake of quanta (*Q*_phar_) resulted in increased photosynthetic rates. The net oxygen evolution rate (*r*_O_) increased by 18% and the fluorescence-based electron flux at PSII (*r*_F_) by 26% (Table 1).

In a next step, the metabolic sink capacity, i.e., the metabolic electron demand, and the photosynthetically provided electrons were calculated to gain a deeper understanding of the cellular sink–source balancing. Table 2 depicts the calculated cellular electron demand for biomass formation and electron fluxes in the electron transfer chain (ETC) under the conditions investigated. The O_2_-based electron supply rate, *r*_O,gross_, was based on *r*_O_ and corrected for the respective measured respiration rate (*r*_O,gross_ = *r*_O_ + *r*_resp_). *r*_O_ and *r*_resp_ were measured at the light intensity applied under the respective cultivation condition. The alternative electron flux (*r*_AEF_) was defined as the difference between oxygen- and fluorescence-based electron fluxes through PSII (*r*_AEF_ = *r*_F_ − *r*_O,gross_). The total electron demand was estimated from the N- and C-assimilation rates. Two assumptions were made: (i) eight additional electrons are required for the assimilation of one molecule of nitrate as compared to ammonium and (ii) a minimum of four electrons is required for the assimilation and reduction of one molecule CO_2_.

**Table 2:** Electron demands and supply in PCC6803 at the investigated sink–source ratios with ammonium or nitrate as N source

	LLHC		HLHC		HLLC	
	NH_4_	NO_3_	NH_4_	NO_3_	NH_4_	NO_3_
*r*_C_	64 ± 10	71 ± 7	232 ± 37	342 ± 49	101 ± 18	111 ± 11
*r*_nitrate_	–	30 ± 4	–	141 ± 24	–	41 ± 6
*r*_D_	64 ± 10	101 ± 11	232 ± 37	483 ± 74	101 ± 18	153 ± 17
*r*_O,gross_	147 ± 6	190 ± 5	417 ± 13	668 ± 17	180 ± 6	203 ± 5
*r*_AEF_	179 ± 17	219 ± 12	626 ± 36	1209 ± 65	92 ± 13	231 ± 16

All rates are given in µmol electrons mg_CDW_^−1^ day^−1^. Mean values and standard deviations were calculated for three different measurement days. See “Materials and methods” for details on the measurements. The cellular electron demand (*r*_D_) was calculated from the electron demand for carbon fixation (*r*_C_) and nitrogen assimilation (*r*_nitrate_). The electron supply rate (*r*_O,gross_) is derived from the sum of *r*_O_ and *r*_Resp_. The alternative electron flux (*r*_AEF_) is calculated by subtracting *r*_O,gross_ from the fluorescence-based electron flux (derived from *r*_F_)

**Fig. 4:**
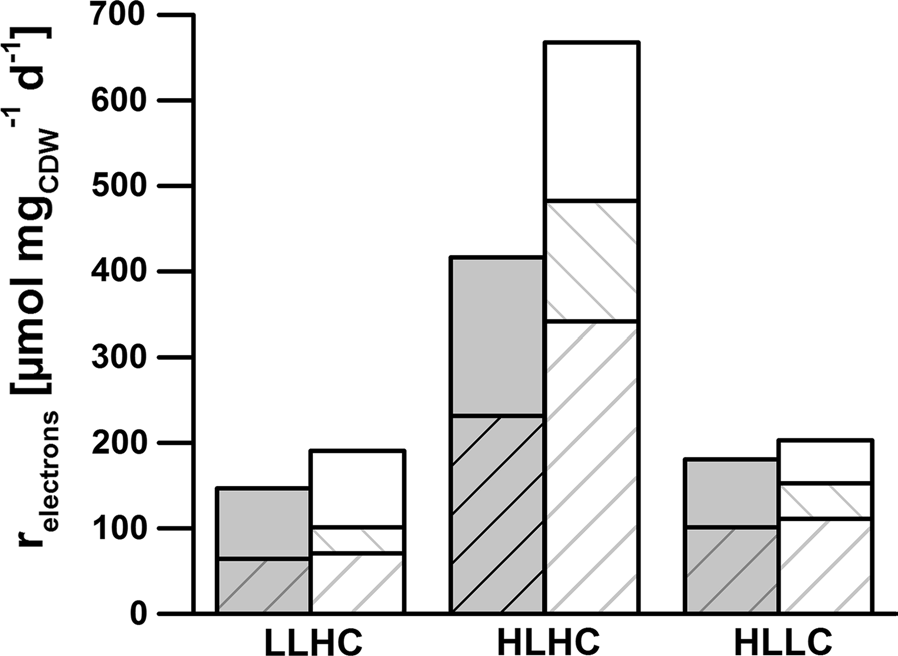
Cellular electron demand and electron supply in PCC6803 under different steady state conditions. Rates are given in µmol electrons mg_CDW_^−1^ day^−1^. PCC6803 was grown with ammonium (gray bars) or nitrate (white bars) as N sources under LLHC (low light high carbon), HLHC (high light high carbon), and HLLC (high light low carbon) conditions. Growth conditions are described in the legend of Fig. 2. Photosystem II electron supply rates are derived from gross O_2_ evolution rates (*r*_O,gross_) and reflected by the entire column heights, whereas electron consumption rates for carbon (striped from lower left to upper right) and nitrate (striped from upper left to lower right) assimilation are shown as shares of the overall supply rates. Mean values and standard deviations were calculated for three different measurement days. See “Materials and methods” for details on the measurements

Under the LLHC condition, nitrate assimilation resulted in a significantly higher cellular electron demand (*r*_D_) of 101 ± 11 µmol electrons mg_CDW_^−1^ day^−1^ compared to 64 ± 10 µmol electrons mg_CDW_^−1^ day^−1^ for ammonium fed cells. Accordingly, the nitrate reduction costs accounted for 16% of the oxygen-based electron supply rate at PSII, *r*_O,gross_. The net increase of *r*_O,gross_ upon nitrate compared to ammonium assimilation (Table 2, Fig. 4) covered the additional net electron demand for nitrate assimilation. However, *r*_O,gross_ was significantly higher than the calculated metabolic demand. This gap is a consequence of the assumption that the cellular electron demand is dominated by carbon and nitrogen assimilation. Other electron-demanding processes, such as phosphor and sulfur assimilation, were neglected. The calculated electron demand thus constitutes a lower boundary. Simultaneously, the alternative electron flux *r*_AEF_ increased by 22% with nitrate instead of ammonium as N source (Table 2). The alternative electron flux is partly used for ATP regeneration. A higher rate thus indicates a higher energy demand of the cells grown on nitrate, e.g., for active ATP-dependent nitrate import into the cells.

In conclusion, the supply of nitrate as an additional electron sink unexpectedly resulted in an increased photosynthetic efficiency enabling an increase in photosynthetic electron supply. This was contrary to the hypothesized decrease in carbon assimilation and biomass formation under light-limited conditions.

### Effect of an additional electron sink under carbon limitation

In strong contrast to the LLHC condition, the HLLC condition does not involve a limitation by light (source), but by CO_2_ (sink). Under such C limitation, the cells have to balance their metabolism by reducing light harvesting and photosynthetic capacities and by dissipation of excess energy. The additional electron demand for nitrate reduction can thus be covered by a higher but still submaximal PSII efficiency and electron supply without further negative effects on biomass formation. Nitrate reduction and biomass formation thus are not expected to compete for energy.

As expected, the growth rate under HLLC conditions was not affected by the additional electron demand for nitrate assimilation (*µ* = 0.77 and 0.74 day^−1^ with ammonium and nitrate, respectively, Table 1). Electron balancing indicated that reduction of nitrate consumed 20% of the PSII-derived electrons (Table 2), which had to be provided in addition to the electrons required for C fixation. The cells met this additional electron demand by a 31% higher effective quantum efficiency at PSII (*Y*(PSII)). This resulted in a strong increase of *r*_F_ by 59% but only a moderate enhancement of *r*_O_ by 12% (Table 1). Consequently, an increase of the alternative electron flux by a factor of 2.5 was observed under nitrate instead of ammonium supply (Table 2). It should be highlighted that nitrate instead of ammonium assimilation was not reflected by an increase in the quantum absorption rate (*Q*_phar_, Table 1), but only by an increased quantum yield *Y*(PSII).

In summary, the results under HLLC conditions met the expectation that an additional electron sink can be covered by the surplus light and thus energy available, without affecting carbon assimilation and cell growth. The cells did not show increased absorption but an increased quantum yield at PSII.

### Effect of an additional electron sink under excess sink and source availability

Under HLHC conditions, the cells can be expected to maximize their carbon fixation capacity and thus growth. Following the hypothesis of a sink-limited photosynthesis [3, 8], the introduction of an additional electron sink should result in an increased activity of the light reaction to cover the additional electron demand, whereas growth should not be affected.

Surprisingly, the cultivation of PCC6803 under HLHC conditions with nitrate as N source resulted in a 16% higher growth rate as compared to ammonium fed cells (Table 1). In contrast to LLHC and HLLC conditions, nitrate instead of ammonium supply resulted in a significant increase of both N- and C-assimilation rates (Fig. 3). Thereby, an enhancement of *Q*_phar_ by 17% was accompanied by a strong increase of *Y*(PSII) by 53% and of the photosynthesis rates *r*_O_ and *r*_F_ by 69 and 80%, respectively (Table 1).

The electron demand was doubled with nitrate instead of ammonium as N source as a consequence of the increased N- and C-assimilation rates and the additional electron demand for nitrate reduction. This electron demand was covered by a strongly increased photosynthetic electron supply and was accompanied by an enhanced alternative electron flux (Table 2). The relative proportion of electrons required for nitrate assimilation (21%) under HLHC was comparable to those under LLHC and HLLC conditions. The C/N ratio in biomass was similar under all conditions tested and did not significantly differ upon nitrate and ammonium supply (Table 1).

Overall, cells of PCC6803 under HLHC conditions responded in an unexpected manner to nitrate assimilation as additional electron sink, i.e., by a distinctive stimulation of growth (and consequently C and N assimilation) and photosynthetic rates. This indicates that metabolic activity is not only regulated by general source (light) and sink availabilities, but also by the type of electron sinks.

## Discussion

### Nitrate assimilation constitutes a significant electron sink in PCC6803

In the present study, the physiological acclimation of the model organism PCC6803 to an additional electron sink was investigated at different source/sink ratios. The availability of source (light) and sink (CO_2_) was modulated to force the cells into differently balanced metabolic states. The assimilation of nitrate was employed as additional electron sink and compared with cultures grown on ammonium as N-source. In contrast to other studies, in which ammonium was demonstrated to have negative and toxic effects in cyanobacteria by causing photodamage at PSII or interfering with the proton gradient in the thylakoid membrane [37–41], we did not observe any negative effect of ammonium up to 50 mM on the growth pattern and phenotype of PCC6803 in shaking flask batch cultivations (see Additional file 2: Figure S2). Nitrate assimilation consumed 16–21% of the photosynthetically produced electrons (based on *r*_O,gross_ and the C/N content of biomass) under all experimental conditions. This corresponds to electron sink rates of 30 ± 4, 141 ± 24, and 41 ± 6 µmol electrons mg_CDW_^−1^ day^−1^ or, in biotechnological terms, 21, 98, and 29 U g_CDW_^−1^ under LLHC, HLHC, HLLC conditions, respectively. These rates are in the range of or exceed most published electron consumption rates of heterologous systems in cyanobacteria [17–24]. To our knowledge, higher electron withdrawal rates were only measured for a NADPH-dependent alkane reduction mediated by an enoate reductase (123 and 246 U g_CDW_^−1^ for the reaction and the electron consumption rates, respectively) [20]. Nitrate assimilation can therefore be considered a strong natural electron sink in PCC6803. Heterologous electron sinks have typically been introduced to establish a product formation concept. However, investigations on physiological responses to and cellular capacities for such biotransformations are largely missing so far [17–19, 21]. The cultivation and analytic setups developed in this study are suitable for this purpose. Such studies will answer the question if the sink effects observed in this study are specific for the assimilation of different N sources or more generally valid for electron sinks including productive recombinant reactions or pathways.

### PCC6803 can easily cope with additional electron sinks

The physiological response of PCC6803 to an additional electron sink may depend on the actual source/sink ratio, to which the cells are exposed under different growth conditions. Source limitation resulted in slow growth combined with a high photosynthetic efficiency. Sink limitation also resulted in slow growth with cells operating at a low photosynthetic efficiency. Source–sink excess conditions resulted in fast growth combined with a moderate photosynthetic efficiency. Thus, the light reaction obviously did not operate at maximum efficiency although the cells were not sink (carbon) limited. This indicates that other cellular processes limit growth rates. These observations are consistent with the source–sink balance hypothesis, stating that photosynthetically active cells balance light absorption efficiency and light reaction performance with other metabolic capacities (e.g., carbon concentration mechanism, Calvin–Benson cycle, anabolism) according to environmental source/sink ratios [3].

The cultivation of PCC6803 with nitrate as an additional electron sink generally resulted in increased effective quantum yields at PSII and light reaction rates irrespective of the sink/source availability (Tables 1 and 2), whereas the absorptivity of the cells (a*_phy_) remained unaffected. Such an increase in the efficiency of the photosynthetic light reaction has previously been observed with either additional electron consuming reactions under HLLC-type conditions (200 µmol photons m^−2^ s^−1^ and ambient CO_2_) [24] or the synthesis and excretion of sucrose under HLHC-type conditions (100 µmol photons m^−2^ s^−1^, 2% CO_2_ enriched air) [29]. Enhanced light reaction efficiency can be expected under HLLC and HLHC conditions, since the photosynthetic activity was not limited by light and thus source availability. Instead, sink availability or other metabolic capacities such as carbon concentration mechanism, Calvin–Benson cycle, or anabolism can be limiting [3, 8]. The increase of *Y*(PSII) as a response to nitrate assimilation under LLHC conditions was unexpected. The light reaction of photosynthesis is commonly assumed to operate at maximum efficiency under conditions of low light and excess carbon availability. Moreover, the results obtained contradict the view that nitrate reduction is a metabolic burden and should be avoided under light-limited conditions [47]. It can be concluded from the presented results that the cells did not run their light reaction at maximum efficiency under LLHC conditions with ammonium as N source. The cells are thus not operating their light reaction at maximum capacity, possibly for adaption to dynamic changes in the availability of resources (such as N sources). This would mean that cells sacrifice their full metabolic potential for a higher robustness in a dynamic environment. The introduction of nitrate as additional electron sink obviously released further photosynthetic potential. It can be hypothesized that this phenomenon is connected to the increase in alternative electron fluxes and thus a changed ATP/NADPH ratio (see below).

**Fig. 5:**
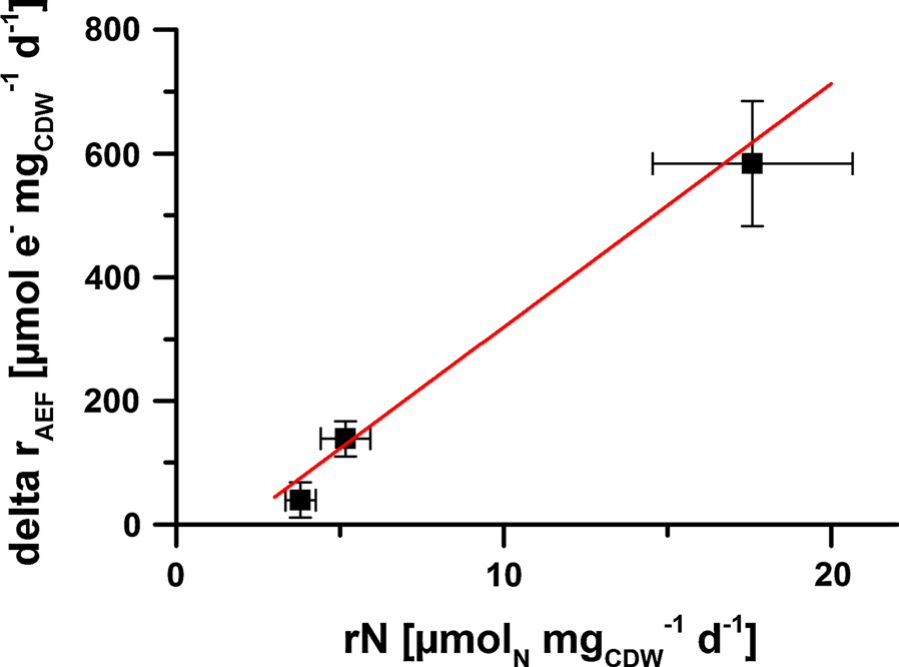
Increase of alternative electron flux (delta *r*_AEF_) in dependence of the nitrate assimilation rate (*r*_N_). Delta *r*_AEF_ corresponds to the difference in alternative electron flux with nitrate and ammonium as N source. See Table 2 for *r*_AEF_’s with the different N sources. Delta *r*_AEF_ is plotted against the respective N assimilation rate (which corresponds to the increased electron demand for nitrate assimilation) under LLHC (low light high carbon), HLHC (high light high carbon), and HLLC (high light low carbon) conditions. The growth conditions are described in the legend of Fig. 2. Mean values and standard deviations were calculated for three different measurement days. See “Materials and methods” for details on the measurements

Another interesting observation was that the alternative electron flux increased in response to nitrate as an additional electron sink under all experimental conditions. This increase correlated linearly with the nitrate assimilation rate (Fig. 5). In principal, the electrons used for the stepwise reduction of nitrate to ammonium are derived from photosynthetic electron transport at the acceptor side of PSI. Therefore, nitrate reduction in cyanobacteria is light-dependent, and the requirement of eight electrons per molecule nitrate results in the evolution of 2 O_2_ molecules at PSII as shown before [48]. Increased O_2_ evolution with nitrate as N-source also was observed in the present study. The alternative electron flux, on the other hand, is calculated from the difference of fluorescence-based and O_2_-based electron flux at PSII. Alternative electron flux is thought to comprise electrons that re-reduce O_2_, e.g., Mehler and Mehler-like reactions. The Mehler-like reaction involves electron transfer via the flavodiiron proteins flv1/flv3 and flv2/flv4 to O_2_ [11, 12, 49, 50]. The flv1/flv3-mediated O_2_ reduction is an electron valve at the acceptor side of PSI. Thus, electrons derived from water splitting at PSII run through the entire photosynthetic electron transport chain (ETC) to end up on O_2_ again. The flavodiiron proteins flv2 and flv4, on the other hand, are discussed to accept electrons from the electron-accepting plastoquinone pocket (*Q*_B_) of PSII [12, 50, 51]. The electron acceptor of flv2/flv4 is not identified, but there are indications that O_2_ can be an electron acceptor at least under carbon limited conditions [52]. With O_2_ as electron acceptor, such electron fluxes over the thylakoid membrane involve the generation of four protons per O_2_ molecule formed on the thylakoid lumen side and the consumption of four protons per O_2_ molecule consumed on the cytoplasmic side. Thereby, and by a possible flux through the ETC as in case of flv1/flv3-mediated O_2_ reduction, such alternative electron flux contributes to ATP synthesis via the proton gradient over the thylakoid membrane. This means that, due to the strong electron consumption for nitrate reduction at the expense of NADPH and the enhanced alternative electron flux, an increase of the ATP/NADPH ratio under conditions with nitrate supply is plausible. The Calvin–Benson cycle requires an ATP/NADPH ratio of 3:2. The linear photosynthetic electron transport does not fulfill this requirement. Therefore, insufficient ATP supply is often limiting carbon assimilation by the Calvin–Benson cycle [53]. Given the increase in alternative electron flux at PSII upon substitution of ammonium with nitrate, the described electron flux over the thylakoid membrane at PSII may, beside cyclic photophosphorylation [3], contribute to the flexible adjustment of the ATP/NADPH ratio. Continuing this argumentation, the ATP/NADPH ratio under ammonium supply would limit the Calvin–Benson cycle, even under source-limited LLHC conditions, and nitrate supply would induce an optimized ATP/NADPH ratio. This could explain, why the cells were able to supply the additionally required electrons for nitrate reduction under LLHC conditions by an increased photosynthesis rate with no negative effect on carbon fixation and growth. To confirm this hypothesis, it would be interesting to investigate on a system level, including transcriptomic and proteomic analyses, how nitrate is capable to stimulate such alternative electron flux and ATP formation and if this phenomenon also is triggered by other artificially introduced electron sinks.

### An electron sink can stimulate light and dark reactions

Another unexpected result was that nitrate instead of ammonium supply under HLHC conditions led to increased C and N assimilation and finally biomass formation rates. This was surprising since the HLHC condition was the condition with most degrees of freedom for metabolic balancing expected to enable growth maximization. The cultures indeed showed high growth rates, but obviously did not exploit their full growth potential with ammonium as N source. As cell growth was not limited by light or carbon supply, the metabolic capacity of the cells obviously differed with the two N sources, with a non-optimum ATP/NADPH ratio as a possible factor limiting metabolic rates and growth in the presence of ammonium (see above). The further stimulation of biomass production and carbon uptake as a result of an additional electron sink also was observed for cyanobacterial strains engineered to synthesize and secrete butanediol [27], sucrose [30] or isoprene [31]. Product formation as an additional sink for electrons and also carbon was proposed to relief a yet unspecified inherent metabolic limitation. The increase of biomass production upon provision of an additional electron sink is a novel observation, but may be explained via the same line of argumentation. Cellular metabolism and particularly the activity of the Calvin–Benson cycle is regulated by the redox state of the cells. It can be hypothesized that electron withdrawal for nitrate reduction lowered the redox potential, e.g., the NAD(P)H/NAD(P) ratio and the oxidation state of the plastoquinone pool. Similar to vascular plants and algae [54], the plastoquinone pool is an important source for redox signals in cyanobacteria with direct impact on the establishment of a certain photo-acclimation state [55, 56]. Accordingly, a more oxidized plastoquinone pool rapidly initiates the transcription of PSII genes and finally increases O_2_ evolution at PSII. Thus, the observed increase in rates for the light reaction as well as the dark reaction upon nitrate supply in HLHC conditions may not only be due to the electron demand for nitrate reduction or a nitrogen source-specific regulation mechanism, but also may reflect a new photo-acclimation state in response to a change in the redox state of the plastoquinone pool.

## Conclusion

Overall, the presented data demonstrated a remarkable potential of PCC6803 to cope with additional electron withdrawal. The results indicate that electron sinks can be beneficial for overall cellular performance. Even a high electron demand can be covered by exploiting inherently available potential of the non-limiting light reaction. Astonishingly, this also was the case under light limiting conditions indicating that the capacity of the light reaction is not necessarily completely exploited under low light conditions. Moreover, an experimental setup was developed in this study, which is suitable to unravel changes in electron demand and electron supply in a quantitative manner. We are convinced that this setup is also applicable to production strains with an inherently high or elevated energy and electron demand.

## Materials and methods

### Bacterial strain and growth conditions

A wild-type *Synechocystis* sp. PCC 6803 strain, in the following referred to as PCC6803, was used in this study. The strain was obtained from the Pasteur Culture Collection of Cyanobacteria (Paris, France) and stored as cryo-culture at − 80 °C in BG11 medium supplemented with 20% (v/v) DMSO.

PCC6803 was cultivated in BG11 or YBG11. For cultivation on solid medium, BG11 according to Rippka et al. [57] was supplemented with 1.5% (w/v) agar and 0.03% (w/v) sodium thiosulfate. A modified YBG11 medium according to Kwon et al. [58] was used for liquid cultivations in shake flasks or bioreactors. For shake flask cultivation, YBG11 was supplemented with 50 mM HEPES and adjusted to pH 8.0 by titration with 10 M NaOH. For continuous bioreactor cultivations, YBG11 was supplemented with 2 mM HEPES and the respective nitrogen source and adjusted to pH 8.0 by titration with 10 M NaOH. Ammonium chloride or sodium nitrate was provided in a concentration of 17.8 mM.

For the bioreactor experiments, PCC6803 was streaked out on BG-11 agar plates from cryo-cultures and cultivated in a plate incubator (poly klima GmbH, Freising, Germany) at 20 µmol photons m^−2^ s^−1^, 30 °C and 75% relative humidity (rH). Emerging colonies were used to inoculate liquid pre-cultures in 250-ml baffled flasks filled with 40 ml YBG-11, which were cultivated in a photoincubator equipped with LED panels (INFORS AG, Bottmingen, Switzerland) at 140 rpm, 30 °C, 50 µmol photons m^−2^ s^−1^ and 75% rH. These cultures were further used to inoculate flat panel bioreactors (Labfors 5 Lux, INFORS AG, Bottmingen, Switzerland) as described below.

### Bioreactor setup and conditions for continuous cultivation

The experiments were conducted in a flat-panel airlift bioreactor system (Labfors 5 Lux, INFORS AG, Bottmingen, Switzerland, Switzerland) with controlled aeration rate (pressurized air or pressurized air enriched with CO_2_), illumination intensity, pH, and temperature. Besides light input, CO_2_ enrichment, and nitrogen source, all environmental parameters were kept constant. The reactors were operated at pH 8.0, controlled by 1 M sodium hydroxide and 15% (v/v) phosphoric acid addition, 30 °C, and 0.5 vvm aeration, which was sufficient for mixing the cell suspension. Light was provided by a LED panel on one side of the reactor (Additional file 3: Figure S3). The other side of the reactor was covered to prevent environmental light to enter the reactor.

The bioreactors were operated continuously, i.e. the biomass concentration was kept constant by providing fresh medium at the same rate as culture broth was removed. The initial dilution rate was set as the growth rate during the batch phase of the cultivation at the respective applied conditions and manually adjusted until the biomass concentration remained stable. The medium was pumped into the reactor with a peristaltic tube pump (IPC-series, ISMATEC, Cole-Parmer GmbH, Wertheim, Germany). The working volume was set to 1.8 l during the cultivations. The filling level was kept constant by pumping culture through a fixed efflux tube at the top of the reactors. The growth rate was derived from the dilution rate according to $D = \mu = \frac{{\dot{V}}}{V}$ with a working volume of *V* = 1.8 l and the feeding rate $\dot{V}.$

Three different conditions were tested for both ammonium and nitrate grown cells: (i) aeration with 1% (v/v) CO_2_ and a low light intensity of 65 µmol photons m^−2^ s^−1^ (LLHC), (ii) aeration with 1% (v/v) CO_2_ and a high light intensity of 250 µmol photons m^−2^ s^−1^ (HLHC), (iii) aeration with air and a high light intensity of 250 µmol photons m^−2^ s^−1^ (HLLC). As soon as the physiological parameters of the cells remained stable for at least a day, the cells were considered to be in a stable metabolic state and were characterized via photon/electron balancing as described below.

### Determination of cell dry weight (CDW) and biomass concentration

OD_750_ was measured in duplicates at least 14 times per steady state in a photo-spectrometer (Libra S12, Biochrom Ltd, Cambridge, Great Britain) at 750 nm. Samples for the determination of the biomass concentration were diluted to an OD_750_ between 0.1 and 0.3. The correlation between OD_750_ and cell dry weight (CDW) concentration was determined for each analyzed metabolic state independently as a mean of three independent measuring days. Fifty microlitre of cell suspension from the reactor were centrifuged (Centrifuge 5810R, rotor FA-45-6-30, Eppendorf AG, Hamburg, Germany) for 10 min at 10 °C and 7000×*g*. The supernatant was discarded and the pellet washed with distilled water. After a second centrifugation step at the same conditions, the cells were resuspended in 0.5 ml of distilled water and transferred to a glass tube. The cells were dried at 70 °C until a constant weight was reached. The weight of the dry biomass was used to calculate the correlation factor between OD_750_ and CDW concentration.

### Estimating cellular composition

Biomass composition was evaluated on the elemental level. The C/N ratio was analyzed in duplicates on three different measuring days for each metabolic state. Fifty microlitre were sampled from the reactor and centrifuged for 10 min at 10 °C and 7000×*g* (Centrifuge 5810R, rotor FA-45-6-30, Eppendorf AG, Hamburg, Germany). The supernatant was discarded and the pellet washed with distilled water. After a second centrifugation step for 10 min at 10 °C and 7000×*g*, the cells were resuspended in 1 ml of distilled water and transferred to an Eppendorf cup. The samples were lyophilized (Freezone 2.5, Labconco, Kansas City, USA) and stored until further measurement. The measurement of the carbon and nitrogen content of the dried biomass was performed with a Vario EL Cube elemental analyzer (Elementar Analysegeräte GmbH, Langenselbold, Germany).

### Determination of the cellular Chl*a* content

The Chl*a* content of the cells was determined spectroscopically for each metabolic state on three different measuring days. Three samples of a defined volume were centrifuged for 10 min at 10,000×*g* and 4 °C (Heraeus Fresco 17, Thermo Scientific, Waltham, USA). The pellet was resuspended in 1.5 ml of ice-cold methanol and homogenized in a bead-mill (Precellys Evolution, BERTIN Technologies, Saint Quentin en Yvelines Cedex, France) for 30 s at 8000 rpm. The homogenized cells were kept on ice for 20 min followed by centrifugation for 10 min at 17,000×*g* and 4 °C (Heraeus Fresco 17, Thermo Scientific, Waltham, USA). The absorption of the extract was measured at 666 nm against a methanol blank. The Chl*a* concentration was calculated using the specific correlation factor of 79.95 l g^−1^ cm^−1^ [59].

### Measurement of the effective quantum yield at PSII

Fluorescence analysis was performed with a Multi-Color PAM [60] (Heinz Walz GmbH, Effeltrich, Germany) to determine the quantum yield at PSII (*Y*(PSII)), i.e. the number of quanta productively used within PSII for charge separation divided by the number of quanta absorbed. This procedure is well established for algae [44, 61]. For cyanobacteria, *Y*(PSII) is considered to be underestimated due to additional fluorescence emission caused by the light harvesting phycobilisomes upon excitation [62–65]. To minimize this underestimation of *Y*(PSII), the analysis was modified, using 400 instead of 625 nm for the measurement light to avoid excitation of the phycobilins. White light was used as actinic light and for the saturation flash. A light induction curve (LIC) was recorded with increasing light intensities. Supernatant from the reactor was used to blank the basal fluorescence of the medium. *Y*(PSII) was interpolated for the light intensity provided in the bioreactor. 9–12 LIC were measured for each metabolic state. The measurements were conducted at least nine times per steady state (minimum three times per measuring day) on three independent measuring days.

### Determination of photosynthetic rates

The photosynthetic capacity and activity of PCC6803 was characterized by determining the cellular quantum absorption rate (*Q*_phar_), the net O_2_ evolution rate (*r*_O_), as well as the maximum electron flux at PSII (*r*_F_) estimated from *Y*(PSII). These parameters were determined as described by Jakob et al. and Wagner et al. [42, 43]. *r*_O_ and *r*_F_ are given as O_2_ evolution rates. *Q*_phar_ is given as a quantum uptake rate.

*Q*_phar_ can be estimated based on the emission spectrum of the LED panel of the reactor (see Additional file 3: Figure S3) and the specific in vivo Chl*a*-specific absorption spectrum of the cells. The theoretical background for the use of the Chl*a*-specific absorption coefficient is described by Blache et al. [66]. The wavelength-dependent Chl*a*-specific absorption coefficient of the cells (see Additional file 4: Figure S4) is defined as

$$a^{*} \left( \lambda \right) = 2.3 \cdot \frac{{{\text{A}}\left( \lambda \right)}}{{d \cdot c\left( {{\text{Chl}}a} \right)}}$$

with the wavelength-dependent absorption A of the sample, the conversion factor 2.3 for the transformation of log_10_ to ln, the path length of the cuvette *d*, and the measured Chl*a* concentration *c*(chl*a*). The absorption spectra of the cells were measured with a dual beam spectrophotometer equipped with an adapter for dispersive samples to correct for light scattering (Zeiss M500, Carl Zeiss AG, Oberkochen, Germany). The absorption spectra were normalized to OD_750_. The emission spectra of the light sources were determined using a spectroradiometer (Tristan 4.0, m-u-t GmbH, Wedel, Germany). These two spectra form the basis for the calculation of the photosynthetically active quantum absorption rate *Q*_phar_ (the wavelength-dependent *Q*_phar_ is depicted in Additional file 5: Figure S5). According to Gilbert et al. [67], *Q*_phar_ was estimated via the following equation:

$$Q_{\text{phar}} = \mathop \int \limits_{{400\;{\text{nm}}}}^{{700\;{\text{nm}}}} Q\left( \lambda \right) - Q\left( \lambda \right) \cdot e^{{ - \left( {a^{*} \left( \lambda \right) \cdot c\left( {{\text{Chl}}a} \right) \cdot d} \right)}} d\lambda$$

with *Q*_phar_ and *Q* standing for the photosynthetically absorbed quanta and the photosynthetically available quanta, respectively.

The net O_2_ evolution rate *r*_O_ in the different metabolic states was measured in triplicates on three different days using a Clark-type electrode (MI-730, Microelectrodes Inc., Bedford, USA). A LIC was performed, determining the O_2_ evolution rates at the cultivation light intensity and respiration rates in the following dark phase (Additional file 1: Figure S1). The net O_2_ formation rate was calculated by correcting the measured O_2_ evolution rate for the corresponding respiration rate.

The maximum electron flux through PSII *r*_F_, expressed as an O_2_ evolution rate, was estimated based on the measured *Y*(PSII) at the applied cultivation light intensity (see above) by applying the following equation:

$$r_{\text{F}} = \frac{{Y\left( {\text{PSII}} \right) \cdot Q_{\text{phar}} \cdot 0.5 \cdot 0.25 \cdot {\text{Chl}}a_{\text{CDW}} }}{{d \cdot c\left( {{\text{Chl}}a} \right)}}$$

with *d* standing for the reactor vessel diameter and Chl*a*_CDW_ for the specific chlorophyll content of the cells based on cell dry weight (CDW). The factors 0.5 and 0.25 account for the assumptions that two quanta are required to feed one electron into the ETC and that four electrons are required to form one molecule of O_2_, respectively.

The photosynthetic quotient PQ was determined for bioreactor samples by measuring the CO_2_ and O_2_ exchange rates in a respirometer (Biometric Systems, Weiterstadt, Germany) as described in Wagner et al. [43]. The carbon fixation rate was derived according to [68] based on PQ and was derived from the following formula:

$$r_{\text{C}} \left( {\upmu{\text{mol}}\;{\text{day}}^{ - 1} \;{\text{mg}}_{\text{CDW}}^{ - 1} } \right) = \frac{{r_{\text{O}} }}{\text{PQ}}.$$

PQ, *Q*_phar_ and *r*_O_ were determined on three independent measuring days.

## Additional files

**Additional file 1: Figure S1.** Light induction curves (LIC) of PCC6803 under different sink–source availabilities. LICs were performed for cells grown in the conditions low light high carbon (LLHC, panel A), high light high carbon (HLHC, panel B), and high light low carbon (HLLC, panel C). See “Materials and methods” section for further experimental details. LIC’s were measured in triplicates on three different measuring days per condition.

**Additional file 2: Figure S2.** Growth of PCC6803 at different ammonium concentrations. No negative effect on growth in batch mode was observed up to an ammonium concentration of 50 mM. PCC6803 was cultivated in shake flasks at 50 µmol photons m^−2^ s^−1^ in YBG11 supplemented with 50 mM HEPES at pH 7.8 and different concentrations of ammonium chloride ranging from 2.5 to 50 mM. Cultures were grown in duplicates.

**Additional file 3: Figure S3.** Emission spectrum of the bioreactor LED panel used in this study.

**Additional file 4: Figure S4.** Chlorophyll-specific absorption spectra of PCC6803 under different sink–source availabilities. The spectra were normalized to the chlorophyll *a* concentration and the OD_750_ of the respective condition. The spectra were measured for cells in the investigated metabolic states low light high carbon (LLHC, panel A), high light high carbon (HLHC, panel B), and high light low carbon (HLLC, panel C). See “Materials and methods” section for further experimental details.

**Additional file 5: Figure S5.** Wavelength-dependent quantum uptake rate (Q_phar_) of PCC6803 under different sink–source availabilities. Spectra were measured on three different days per condition and averaged: low light high carbon (LLHC, panel A), high light high carbon (HLHC, panel B), and high light low carbon (HLLC, panel C).

## Abbreviations

*µ*: growth rate (day^−1^)
CDW: cell dry weight
ETC: electron transport chain
*Y*(PSII): effective quantum yield at photosystem II at the given light intensity
*Q*_phar_: the rate of absorbed quanta by the cells (mmol quanta mg_CDW_^−1^ day^−1^)
*r*_O_: net O_2_ evolution rate (µmol O_2_ mg_CDW_^−1^ day^−1^)
*r*_F_: maximum electron flux at PSII estimated from the effective fluorescence yield around PSII (µmol O_2_ mg_CDW_^−1^ day^−1^)
PAR: photosynthetically accessible radiation (400–700 nm)
Chl*a*: chlorophyll *a*
