# Supplementary material for: Electron balancing under different sink conditions reveals positive effects on photon efficiency and metabolic activity of Synechocystis sp. PCC 6803
Source: Biotechnol Biofuels. 2019 Feb 27;12:43. doi: 10.1186/s13068-019-1378-y (PMC6391784; doi:10.1186/s13068-019-1378-y)
Supplement: Supplementary file 1 — Additional file 1: Figure S1. Light induction curves (LIC) of PCC6803 under different sink–source availabilities. LICs were performed for cells grown in the conditions low light high carbon (LLHC, panel A), high light high carbon (HLHC, panel B), and high light low carbon (HLLC, panel C). See “Materials and methods” section for further experimental details. LIC’s were measured in triplicates on three different measuring days per condition. [file 13068_2019_1378_MOESM1_ESM.docx]

**Figure S1:** Light induction curves (LIC) of PCC6803 under different sink-source availabilities. LICs were performed for cells grown in the conditions low light high carbon (LLHC, panel A), high light high carbon (HLHC, panel B), and high light low carbon (HLLC, panel C). See Materials and Methods section for further experimental details.
