# Supplementary material for: Electron balancing under different sink conditions reveals positive effects on photon efficiency and metabolic activity of Synechocystis sp. PCC 6803
Source: Biotechnol Biofuels. 2019 Feb 27;12:43. doi: 10.1186/s13068-019-1378-y (PMC6391784; doi:10.1186/s13068-019-1378-y)
Supplement: Supplementary file 2 — Additional file 2: Figure S2. Growth of PCC6803 at different ammonium concentrations. No negative effect on growth in batch mode was observed up to an ammonium concentration of 50 mM. PCC6803 was cultivated in shake flasks at 50 µmol photons m−2 s−1 in YBG11 supplemented with 50 mM HEPES at pH 7.8 and different concentrations of ammonium chloride ranging from 2.5 to 50 mM. Cultures were grown in duplicates. [file 13068_2019_1378_MOESM2_ESM.docx]

**Figure S2:** Growth of PCC6803 at different ammonium concentrations. No negative effect on growth in batch mode was observed up to an ammonium concentration of 50 mM. PCC6803 was cultivated in shake flasks at 50 µmol photons m^-2^ s^-1^ in YBG11 supplemented with 50 mM HEPES at pH 7.8 and different concentrations of ammonium chloride ranging from 2.5 to 50 mM.
