# Supplementary material for: Electron balancing under different sink conditions reveals positive effects on photon efficiency and metabolic activity of Synechocystis sp. PCC 6803
Source: Biotechnol Biofuels. 2019 Feb 27;12:43. doi: 10.1186/s13068-019-1378-y (PMC6391784; doi:10.1186/s13068-019-1378-y)
Supplement: Supplementary file 3 — Additional file 3: Figure S3. Emission spectrum of the bioreactor LED panel used in this study. [file 13068_2019_1378_MOESM3_ESM.docx]

**

**

**Figure S3:** Emission spectrum of the bioreactor LED panel used in this study normalized to 1 µmol photons m^-2^ s^-1^ PAR.
