# Supplementary material for: Electron balancing under different sink conditions reveals positive effects on photon efficiency and metabolic activity of Synechocystis sp. PCC 6803
Source: Biotechnol Biofuels. 2019 Feb 27;12:43. doi: 10.1186/s13068-019-1378-y (PMC6391784; doi:10.1186/s13068-019-1378-y)
Supplement: Supplementary file 4 — Additional file 4: Figure S4. Chlorophyll-specific absorption spectra of PCC6803 under different sink–source availabilities. The spectra were normalized to the chlorophyll a concentration and the OD750 of the respective condition. The spectra were measured for cells in the investigated metabolic states low light high carbon (LLHC, panel A), high light high carbon (HLHC, panel B), and high light low carbon (HLLC, panel C). See “Materials and methods” section for further experimental details. [file 13068_2019_1378_MOESM4_ESM.docx]

**Figure S4:** Chlorophyll *a*-specific absorption spectra of PCC6803 under different sink-source availabilities. The spectra were measured for cells in the investigated metabolic states low light high carbon (LLHC, panel A), high light high carbon (HLHC, panel B), and high light low carbon (HLLC, panel C). See Materials and Methods section for further experimental details.
