# Supplementary material for: Electron balancing under different sink conditions reveals positive effects on photon efficiency and metabolic activity of Synechocystis sp. PCC 6803
Source: Biotechnol Biofuels. 2019 Feb 27;12:43. doi: 10.1186/s13068-019-1378-y (PMC6391784; doi:10.1186/s13068-019-1378-y)
Supplement: Supplementary file 5 — Additional file 5: Figure S5. Wavelength-dependent quantum uptake rate (Qphar) of PCC6803 under different sink–source availabilities. Spectra were measured on three different days per condition and averaged: low light high carbon (LLHC, panel A), high light high carbon (HLHC, panel B), and high light low carbon (HLLC, panel C). [file 13068_2019_1378_MOESM5_ESM.docx]

**Figure S5:** Wavelength-dependent quantum uptake rate (Q_phar_) of PCC6803 under different sink-source availabilities. Spectra were measured on three different days per condition and averaged: low light high carbon (LLHC, panel A), high light high carbon (HLHC, panel B), and high light low carbon (HLLC, panel C).
